# Spatial clusters of HIV-1 genotypes in a recently infected population in Yunnan, China

**DOI:** 10.1186/s12879-019-4276-9

**Published:** 2019-07-29

**Authors:** Min Chen, Yanling Ma, Huichao Chen, Jie Dai, Hongbing Luo, Chaojun Yang, Lijuan Dong, Xiaomei Jin, Min Yang, Li Yang, Lijun Song, Zhizhong Song, Manhong Jia

**Affiliations:** Institute for AIDS/STD Control and Prevention, Yunnan Center for Disease Control and Prevention, No. 158, Dongsi Street, Xishan District, Kunming, 650022 Yunnan Province China

**Keywords:** Human immunodeficiency virus-1 (HIV-1), Genetics, Recent infections, Spatial cluster, China

## Abstract

**Background:**

As a gateway for HIV-1 in China, Yunnan has experienced dramatic changes in HIV-1 epidemics, during which HIV-1 genotypes have become complex. To track dynamic changes in HIV-1 genotypes, an HIV-1 molecular epidemiological study was implemented in the recently infected population in Yunnan.

**Methods:**

From 6,357 HIV-1-positive samples diagnosed during the first half of 2015 in Yunnan, 586 samples were identified as recent infections with BED-capture enzyme immunoassay (CEIA) and were subjected to phylogenetic analyses. Spatial scanning analyses for the main HIV-1 genotypes were also performed.

**Results:**

Among the 439 specimens successfully genotyped, more than ten genotypes were detected, including CRF08_BC (45.3%), CRF07_BC (19.4%), unique recombinant forms (URFs) (18.2%), CRF01_AE (11.4%), subtype C (2.1%), CRF85_BC (1.1%), CRF55_01B (0.9%), subtype B (0.5%), CRF64_BC (0.5%), CRF59_01B (0.2%), CRF83_cpx (0.2%) and CRF87_cpx (0.2%). Females, Chinese, heterosexual contact and intravenous drug injection were significantly associated with CRF08_BC infection; homosexual contact was significantly associated with CRF01_AE and CRF07_BC infection; males and non-Chinese had a higher risk of URF infection than females. Among all HIV-1 genotypes, the geographic coverage of CRF08_BC was the largest. For CRF08_BC, CRF07_BC, URFs and CRF01_AE, spatial clusters were detected. The two CRF08_BC clusters and one URF cluster were associated with heterosexual transmission, and two of CRF01_AE clusters were associated with homosexual transmission. Transmitted drug resistance (TDR)-associated mutations were detected in 2.4% of individuals.

**Conclusions:**

The diversity of HIV-1 genotypes increased in recent infections because of a long-term HIV-1 epidemic in Yunnan. The predominant HIV-1 strains showed distinct demographic characteristics and formed spatial clusters. These findings improved our understanding of the evolution of HIV-1 in Yunnan and provided information for further HIV-1 control and prevention.

**Electronic supplementary material:**

The online version of this article (10.1186/s12879-019-4276-9) contains supplementary material, which is available to authorized users.

## Background

Since HIV-1 was first identified among intravenous drug users (IDUs) in western Yunnan in 1989, HIV-1 epidemics have dramatically increased in Yunnan [1–3]. Yunnan is considered the HIV-1 gateway in China. Originating in Yunnan, different HIV-1 genotypes have spread to other parts of China [1, 4]. Over the past nearly 30 years, unprotected sexual contact has replaced intravenous drug injection as the predominant route of HIV-1 transmission [2]. By the end of 2014, the number of people living with HIV/AIDS (PLWHA) in Yunnan was 80,610 (including 50,426 people living with HIV and 30,184 AIDS patients), which was the highest among all provinces in China. Among all PLWHA in Yunnan, 68.9% received antiretroviral therapy, which was higher than the national average (63.2%). Among the annual newly reported cases, the proportion of cases attributed to intravenous drug use decreased from 28.6% in 2009 to 9.3% in 2014; the proportion of heterosexually infected cases increased from 62.5% in 2009 to 85.1% in 2014, while the proportion of homosexually infected cases increased from 1.4% in 2009 to 4.3% in 2014.

Initially, subtype B and subtype C emerged among IDUs [5–7], and CRF01_AE was found among female sex workers (FSWs) [8]. In the 1990s, CRF07_BC and CRF08_BC formed through the recombination of subtype B and subtype C, which were circulating among IDUs in Yunnan and transmitted along drug trafficking routes [9, 10]. Through the populations with the dual risk factors of intravenous drug use and heterosexual contact [11], CRF08_BC and CRF07_BC emerged in the heterosexually transmitted population [12]. In the late 2000s, CRF08_BC became the chief HIV-1 genotype among both heterosexuals and IDUs, and the composition of HIV-1 genotypes was nearly the same in these two populations [12]. The first HIV-positive man who had sex with men (MSM) in Yunnan was reported in 2004. The surveillance showed that CRF01_AE and CRF07_BC were the main strains among MSM, accounting for more than 90% of cases [13]. Recently, CRF55_01B and CRF59_01B were also detected in MSM in Yunnan [14], which were first identified among MSM in southern China and northeastern China, respectively [15, 16]. Moreover, the spatial distribution of HIV-1 genotypes dynamically changed. The main HIV-1 genotypes spread from the original areas to other areas of Yunnan. For example, CRF01_AE was transmitted from western to eastern Yunnan, and CRF08_BC and CRF07_BC were transmitted from southeast of Yunnan to other areas.

The genetic diversity of HIV-1 is striking in Yunnan. In addition to the abovementioned subtypes and circulating recombinant forms (CRFs), a multitude of URFs were also detected in Yunnan [12, 17]. In the late 2000s, the proportion of URFs ranked second only to that of CRF08_BC in recent infections [12]. These URFs contributed to the formation of novel CRFs. In recent years, at least nine CRFs were identified in western Yunnan [18–25]. These CRFs consist of components from subtype B, subtype C and CRF01_AE. A respective phylogenetic study showed that some of these CRFs originated in the early 1990s [9].

With the development of HIV-1 epidemics, the demographic profile of those infected has changed, while the characteristics and distribution of HIV-1 genotypes have also changed. To track the changing trend of HIV-1 genotypes in Yunnan, we performed an HIV-1 molecular epidemiological study among a recently infected population in Yunnan in 2015.

## Methods

### Study participants and sample collection

From January 2015 to June 2015, 6357 HIV/AIDS cases were newly reported through different screening methods in Yunnan Province. Before carrying out the recent HIV-1 infection assay, 1755 long-term infected cases with CD4^+^ T lymphocytes < 200 cells/μl or AIDS-defining illnesses were excluded. The remaining 4602 cases were further tested for the status of recent HIV-1 infection. The adults’ written consents were provided by themselves. The juveniles’ consents for HIV testing were provided by their guardians, if they had HIV, written consents about this study for HIV control and prevention were further obtained from their guardians when informing testing results. The study was approved by the Biomedical Ethics Review Committee of Yunnan Province.

### Recent HIV-1 infection tested with BED-capture enzyme immunoassay (CEIA)

BED-capture enzyme immunoassay (BED-CEIA) (Calypte Biomedical Corporation, Portland, OR, USA) was used to test for the recency of HIV-1 infection. According to the manufacturer’s instructions, plasma specimens were first tested individually. If the normalized OD (ODn) was > 1.2, the specimen was classified as having a long-term infection. Specimens with ODn < 1.2 were tested again in triplicate for confirmation. In confirmatory testing, specimens with ODn < 0.8 were classified as recent infections. According to the evaluation carried out in China, the duration of a recent HIV infection determined by BED-CEIA was 168 days after seroconversion.

### Amplification of HIV-1 gene fragments

According to the manufacturer’s instructions, viral RNA was extracted from 140 μl of plasma using the QIAamp Viral RNA Mini Kit (Qiagen, Valencia, CA, USA). The partial *gag* gene (HXB2: 781–1861), partial *pol gene* (HXB2: 2147–3462) and partial *env* gene (HXB2: 7002–7541) were amplified using nested polymerase chain reactions (PCR). The successfully amplified products were sent to SinoGenoMax Co. (Beijing, China) for sequencing. The primers and procedures for nested PCR and the primers for sequencing were described in a previous study [13].

### Sequence analysis

The sequences were assembled using Sequencher 5.1 (Gene Codes, Ann Arbor, MI). The assembled sequences were aligned with Bio-Edit 7.0 software and further manually edited. HIV-1 reference sequences were selected and downloaded from the HIV databases of the Los Alamos National Laboratory (LANL) (https://www.hiv.lanl.gov). Neighbour-joining phylogenetic trees were construed with the Kimura 2-parameter model with 1000 bootstrap replicates with MEGA version 6.0 [26]. The sequences with possible intersubtype recombination were analysed with the Recombination Identification Programme (RIP, version 3.0; https://www.hiv.lanl.gov). HIV-1 genotyping was based on at least two segments of the *gag*, *pol* and *env* genes. Samples with only one gene segment were excluded. Finally, 260 samples had *gag*, *pol* and *env* segments, 108 had *gag* and *pol* segments, 45 had *gag* and *env* segments, and 26 had *pol* and *env* segments. The above 439 samples were considered to have achieved reliable HIV-1 genotyping.

### Geographic distribution analysis and spatial scan statistics analysis of HIV-1 genotypes

The distribution density of each HIV-1 genotype at the county level was displayed using a dot density map, within which the number of dots in a county represented the proportion of cases with a given genotype in this county among all the cases successfully genotyped. One dot represented 0.025% of the population.

The spatial scanning statistic is used to detect possible spatial clusters by scanning across space with a radius varying circular window. The null hypothesis is that the risks of event occurrence are equal between the inside and outside windows, while the alternative hypothesis is that the risks are different between the inside and outside windows.

Under the Poisson assumption, the log likelihood ratio (LLR) is calculated as:

$$ LLR={\left(\frac{c}{E\left[c\right]}\right)}^c{\left(\frac{C-c}{C-E\left[c\right]}\right)}^{C-c}I\left(\right) $$

where C is the total number of cases; c is the observed number of cases within the window; E [c] is the covariate adjusted expected number of cases within the window under the null-hypothesis; I() is an indicator function.

For each location, by varying the size, a window with the maximum likelihood will be found. The *p*-value is obtained through Monte Carlo simulations, by comparing the rank of the maximum likelihood from the real data set with those from random simulated data sets. If this rank is R, then *p* = R / (1+ n), where n is the number of random simulations. Among the regions with statistical significance, the regions with the maximum likelihood are defined as the most likely cluster (primary cluster), and the others with a smaller likelihood are defined as secondary clusters.

The relative risk (RR) represents the aggregation risk of the cluster compared with the rest of the regions. RR is defined as the estimated risk within the cluster divided by the estimated risk outside the cluster. The mathematical notation is as follows:

$$ RR=\frac{c/E\left[c\right]}{\left(C-c\right)/\left(C-E\left[c\right]\right)} $$

where the meaning of the symbols are the same as above.

In this study, SaTScan 9.6 was used for the spatial scan statistics analysis [27]. A Poisson-based model combined with circular scan windows was used. For the analysis of each HIV-1 genotype, three types of files (case file, population file and coordinates file) were submitted into SaTScan. The case file contained the numbers of cases with a given HIV-1 genotype in each county. The population file contained the number of populations in each county. The coordinates file contained the coordinates of each county. The most commonly used maximum spatial cluster size is 50% of the population at risk [28–34]. To choose the maximum spatial cluster size, a sensitivity analysis was performed with 10, 30 and 50% of the population at risk in the spatial window (Additional file 1: Table S1). The significant clusters of CRF01_AE, CRF07_BC, CRF08_BC and URFs showed no difference when using these settings. Therefore, 10% of the population at risk was used as the maximum spatial cluster size. The spatial clusters were mapped using Quantum GIS [35].

### Genotypic analysis of HIV-1 drug resistance

Surveillance drug resistance mutations (SDRMs) within the obtained *pol* sequences were screened using the Calibrated Population Resistance (CPR) Tool (Version 6.0) on Stanford HIV Drug Resistance Database (http://hivdb.stanford.edu) [36].

### Statistical analysis

Statistical analyses were performed with the SPSS 21.0 statistical analysis software package (SPSS Inc. Chicago, IL). Demographic characteristics associated with HIV-1 genotypes were first analysed with univariate logistic regression. For each HIV-1 genotype, variables marginally significant with *p* < 0.10 in univariate logistic analysis were further analysed with multivariate logistic regression. The trend chi-square test was performed with the linear-by-linear association method under the chi-square test of SPSS. All tests were two-tailed and statistical significance was considered when *p-* value < 0.05.

## Results

### Demographic characteristics of the study participants

**Fig. 1:**
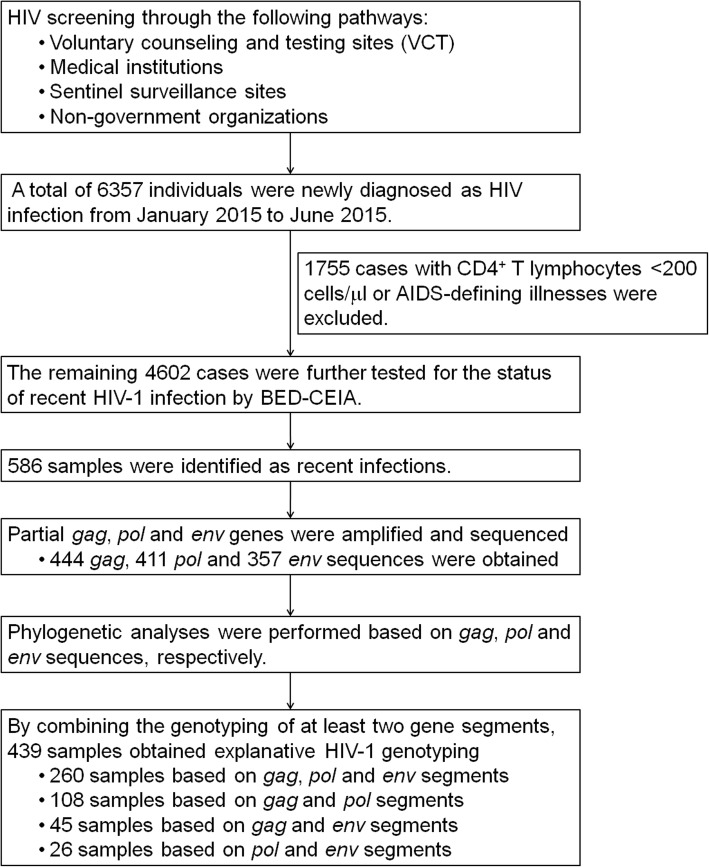
Flow chart of the study process. The flow chart shows the overall study process, including HIV-1 screening and confirmation, recent infection testing and HIV-1 genotyping

As shown in Fig. 1, during the first half of 2015, 6,357 HIV/AIDS cases were newly diagnosed through the different screening methods in Yunnan Province. Based on information regarding disease progression and a BED-CEIA assay, 586 samples were identified as recent infections and were subjected to HIV-1 gene amplification. Finally, 444 *gag*, 411 *pol* and 357 *env* sequences were obtained, which were further subjected to phylogenetic analyses (Additional file 2: Figure S1, Additional file 3: Figure S2, Additional file 4: Figure S3). By combining the genotyping of at least two gene segments, 439 samples obtained explanative HIV-1 genotyping. The constituent of the 439 participants showed no significant differences with that of the total 586 recently infected individuals (Additional file 5: Table S2).

Of the 439 participants, 62.0% (272/439) were males, and 38.0% (167/439) were females; the median age was 35 years (range: 15–81 years); Han ethnicity accounted for 62.0% (272/439), while other ethnicities accounted for 38.0%, including Yi, Hani, Dai, Zhuang, Jingpo, Bai, Hui, Wa, Lahu, Lisu, Miao, Yao, Man, Achang, Buyi, Naxi and Nu. Regarding marital status, 33.7% (148/439) were single, 43.7% (192/439) were married, and 22.6% (99/439) were divorced or widowed. Of the 439 participants, 5.0% (22/439) were non-Chinese, most of which were Burmese living Dehong Prefecture (72.7%, 16/22). For the transmission routes, heterosexual contact, homosexual contact and intravenous drug injection accounted for 81.1% (356/439), 11.2% (49/439) and 7.7% (34/439), respectively.

### HIV-1 genotypes in recent HIV infections

Among the 439 samples successfully genotyped, more than ten HIV-1 genotypes were identified, including two subtypes, nine CRFs and six discrete URFs. CRF08_BC was the most common (45.3%, 199/439), followed by CRF07_BC (19.4%, 85/439), URFs (18.2%, 80/439), CRF01_AE (11.4%, 50/439), subtype C (2.1%, 9/439), CRF85_BC (1.1%, 5/439), CRF55_01B (0.9%, 4/439), subtype B (0.5%, 2/439), CRF64_BC (0.5%, 2/439), CRF59_01B (0.2%, 1/439), CRF83_cpx (0.2%, 1/439) and CRF87_cpx (0.2%, 1/439). Among the six discrete URFs, BC recombinants were the predominant recombinant form (66.3%, 53/80), followed by BC/CRF01_AE (10.0%, 8/80), CRF07_BC/CRF01_AE (8.8%, 7/80), B/CRF01_AE (6.3%, 5/80), C/CRF01_AE (5.0%, 4/80) and CRF07_BC/CRF08_BC (3.8%, 3/80).

### Demographic characteristics associated with HIV-1 genotypes

Demographic characteristics associated with HIV-1 genotypes were further analysed using logistic regression (Table 1). The results of the multivariate analyses showed that homosexual contact was significantly associated with CRF01_AE and CRF07_BC infection; females, Chinese, heterosexual contact and intravenous drug injection were significantly associated with CRF08_BC infection; males and non-Chinese had a higher risk of URF infection than females.

**Table 1:** Demographic characteristics associated with HIV-1 genotypes

	Total	Subjects with given HIV-1 genotype	Univariate analysis		Multivariate analysis	
			*p*	OR (95% CI)	*p*	OR (95% CI)
CRF01_AE						
Gender						
Female	167	12	–	1.000	–	1.000
Male	272	38	0.033	2.098 (1.063~4.140)	0.431	1.356 (0.636~2.889)
Age			0.256			
≤ 30	174	25	–	1.000		
31–50	182	16	0.102	0.574 (0.295~1.117)		
≥ 51	83	9	0.437	0.725 (0.322~1.632)		
Nationality						
Chinese	417	49	–	1.000		
Non-Chinese	22	1	0.320	0.358 (0.047~2.718)		
Race/ethnicity						
Han	272	31	–	1.000		
Others	167	19	0.995	0.998 (0.544~1.831)		
Marital Status			0.034		0.706	
Married	192	15	–	1.000	–	1.000
Divorced/Widowed	99	10	0.510	1.326 (0.573~3.070)	0.727	1.166 (0.493~2.754)
Unmarried	148	25	0.012	2.398 (1.215 (4.735)	0.404	1.392 (0.640~3.026)
Infection Routes			< 0.001		0.001*	
Heterosexual contact	354	32	–	1.000	–	1.000
Homosexual contact	49	18	< 0.001	5.843 (2.945~11.591)	< 0.001*	4.469 (2.016~9.908)
Intravenous drug injection	36	0	0.998	–	0.998	–
CRF07_BC						
Gender						
Male	272	55	–	1.000		
Female	167	30	0.562	0.864 (0.527~1.415)		
Age			0.004		0.165	
≥ 51	83	10	–	1.000	–	1.000
31–50	182	28	0.473	1.327 (0.612~2.878)	0.516	1.305 (0.585~2.908)
≤ 30	174	47	0.009	2.702 (1.288~5.667)	0.092	2.136 (0.884~5.158)
Nationality						
Chinese	417	81	–	1.000		
Non-Chinese	22	1	0.106	0.189 (0.025~1.423)		
Race/ethnicity						
Han	272	60	–	1.000	–	1.000
Others	167	25	0.070	0.622 (0.373~1.039)	0.098	0.630 (0.364~1.089)
Marital Status			0.003		0.820	
Divorced/Widowed	99	14	–	1.000	–	1.000
Married	192	29	0.826	1.080 (0.542~2.153)	0.810	1.090 (0.538~2.210)
Unmarried	148	42	0.010	2.406 (1.232~4.696)	0.545	1.283 (0.573~2.871)
Infection Routes			< 0.001		0.014*	
Heterosexual contact	354	58	–	1.000	–	1.000
Intravenous drug injection	36	6	0.965	1.021 (0.407~2.563)	0.960	1.025 (0.397~2.642)
Homosexual contact	49	21	< 0.001	3.828 (2.035~7.201)	0.004*	2.767 (1.392~5.501)
CRF08_BC						
Gender						
Male	272	103	–	1.000	–	1.000
Female	167	96	< 0.001	2.219 (1.498~3.285)	0.012*	1.760 (1.132~2.738)
Age			0.001		0.216	
≤ 30	174	60	–	1.000	–	1.000
31–50	182	93	0.002	1.985 (1.296~3.042)	0.118	1.512 (0.900~2.540)
≥ 51	83	46	0.002	2.362 (1.385~4.029)	0.121	1.696 (0.870~3.308)
Nationality						
Non-Chinese	22	1	–	1.000	–	1.000
Chinese	417	198	0.004	18.986 (2.531~142.450)	0.004*	21.061 (2.657~166.944)
Race/ethnicity						
Han	272	126	–	1.000		
Others	167	73	0.594	0.900 (0.611~1.326)		
Marital Status			< 0.001		0.807	
Unmarried	148	47	–	1.000	–	1.000
Married	192	99	< 0.001	2.288 (1.463~3.577)	0.809	1.071 (0.614~1.869)
Divorced/Widowed	99	53	0.001	2.476 (1.464~4.186)	0.535	1.229 (0.641~2.356)
Infection Routes			< 0.001		< 0.001*	
Homosexual contact	49	3	–	1.000	–	1.000
Intravenous drug injection	36	14	0.001	9.7548 (2.538~37.507)	< 0.001*	14.443 (3.452~60.437)
Heterosexual contact	354	182	< 0.001	16.225 (4.954~53.138)	< 0.001*	11.029 (3.250~37.429)
URFs						
Gender						
Female	167	23	–	1.000	–	1.000
Male	272	57	0.060	1.660 (0.979~2.815)	0.035*	1.853 (1.043~3.292)
Age						
≤ 30	174	33	–	1.000		
31–50	182	33	0.840	0.946 (0.554~1.615)		
≥ 51	83	14	0.684	0.867 (0.436~1.725)		
Nationality						
Chinese	417	64	–	1.000	–	1.000
Non-Chinese	22	16	< 0.001	14.708 (5.546~39.007)	< 0.001*	17.719 (5.624~55.829)
Race/ethnicity						
Han	272	42	–	1.000		
Others	167	38	0.055	1.613 (0.989~2.630)		
Marital Status			0.601			
Unmarried	148	25	–	1.000		
Married	192	29	0.424	1.254 (0.720~2.186)		
Divorced/Widowed	99	16	0.880	0.948 (0.477~1.884)		
Infection Routes			0.063		0.189	
Homosexual contact	49	5	–	1.000	–	1.000
Heterosexual contact	354	64	0.177	1.942 (0.741~5.091)	0.117	2.208 (0.821~5.936)
Intravenous drug injection	36	11	0.023	3.872 (1.207~12.420)	0.783	1.221 (0.296~5.038)

*: statistical significance

### Spatial distribution of HIV-1 genotypes

**Fig. 2:**
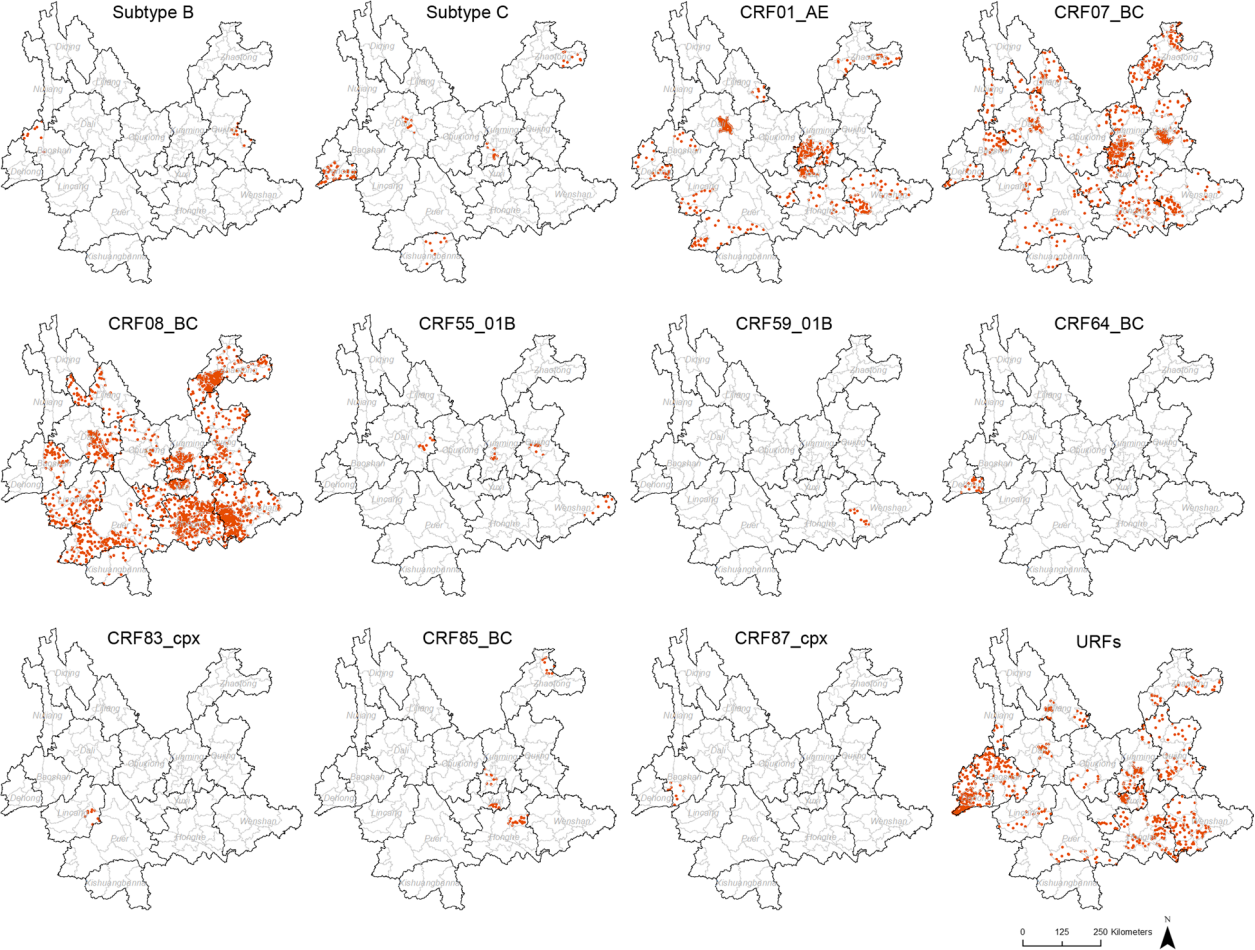
Geographic distribution of HIV-1 genotypes in recent infections in Yunnan. The dot density map for subtype B, subtype C, CRF01_AE, CRF07_BC, CRF08_BC, CRF55_01B, CRF55_01B, CRF64_BC, CRF83_cpx, CRF85_BC, CRF87_cpx and URFs shows the percentage of each genotype in each county. One dot was defined as 0.025% of the population

The spatial distribution of HIV-1 genotypes in recent infections was analysed at the county level (Fig. 2). CRF08_BC was the most widely distributed genotype, which was found in 54.3% (70/129) of the counties in Yunnan Province. However, more CRF08_BC cases were found in the six eastern prefectures (Zhaotong, Qujing, Kunming, Yuxi, Honghe and Wenshan). The other three main genotypes, CRF07_BC, URFs and CRF01_AE, were found in 38.0% (49/129), 34.1% (44/129) and 20.2% (26/129) of all counties, respectively. The distributions of subtype C, CRF85_BC, CRF55_01B, subtype B, CRF64_BC, CRF59_01B, CRF83_cpx and CRF87_cpx were limited.

**Fig. 3:**
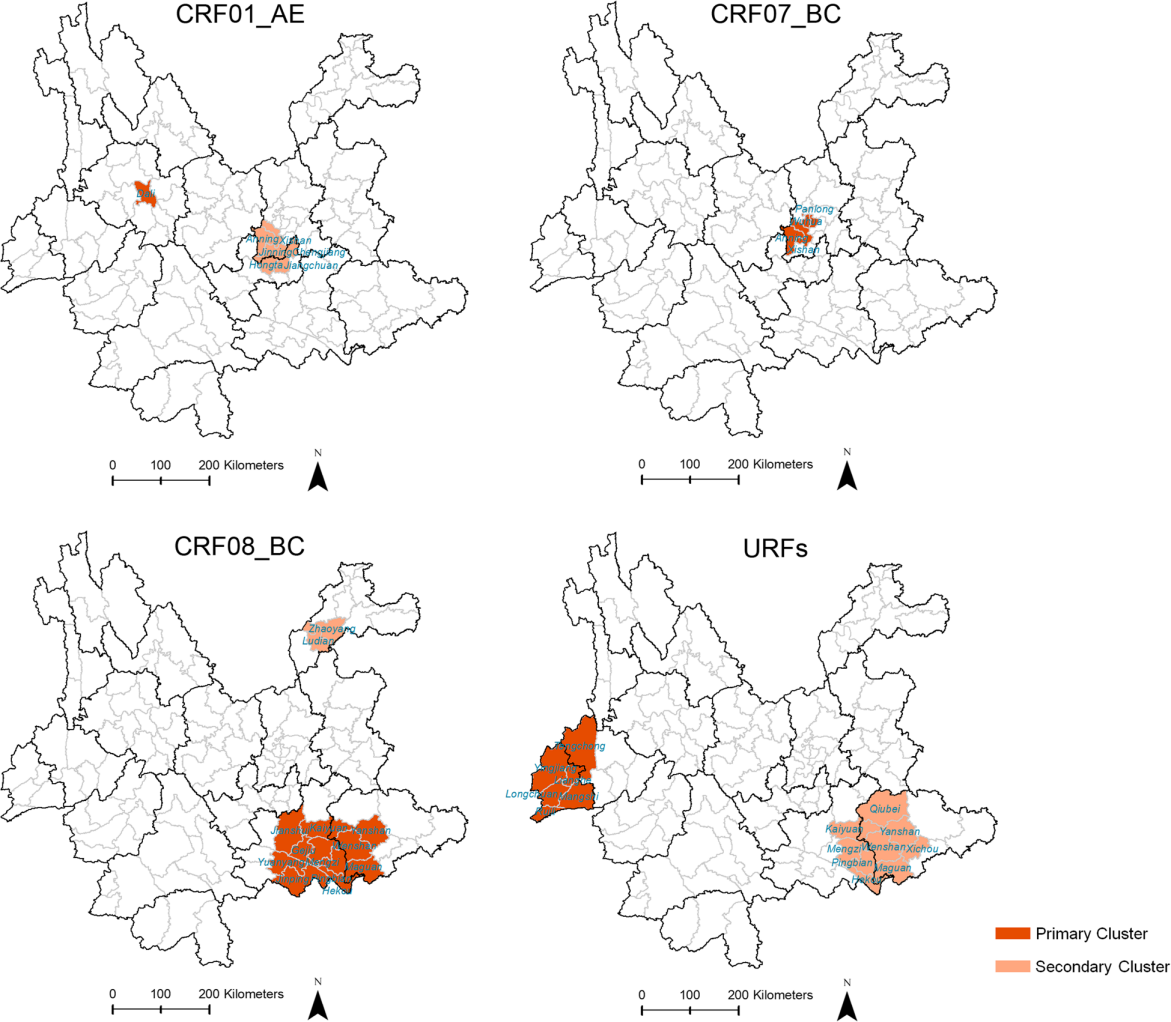
Spatial clusters of HIV-1 genotypes in recent infections in Yunnan. The counties in the primary and secondary clusters of CRF01_AE, CRF07_BC, CRF08_BC and URFs are marked in the maps

Spatial clusters were further analysed using spatial scan statistics (Fig. 3). For CRF08_BC, two non-overlapping statistically significant clusters were detected. Among them, the most likely cluster (RR = 4.78) included 11 counties in Honghe Prefecture and Wenshan Prefecture, and the secondary clusters (RR = 4.24) included two counties in Zhaotong Prefecture. In these two spatial clusters, 95.2 and 90.0% of observed cases were infected through heterosexual contact (Table 2). For CRF07_BC, there was only one most likely cluster (RR = 3.68) detected in Kunming Prefecture (Table 2). For CRF01_AE, two non-overlapping statistically significant clusters were detected. Among them, the most likely cluster (RR = 17.41) was located in one county in Dali Prefecture, and the secondary cluster (RR = 5.36) included six counties in Kunming Prefecture and Yuxi Prefecture. In the most likely cluster and the secondary cluster, 80.0 and 63.6% of observed cases were infected through homosexual contact (Table 2). For URFs, two non-overlapping statistically significant clusters were detected. Among them, the most likely cluster (RR = 9.62) was located in the west, including six counties in Dehong Prefecture and Baoshan Prefecture, in which 60.9% of observed cases were infected through intravenous drug use; the secondary cluster (RR = 3.51) was located in the southeast and included nine counties in Honghe Prefecture and Wenshan Prefecture, in which 100.0% of observed cases were infected through heterosexual contact (Table 2).

**Table 2:** The characteristics of spatial clusters for CRF01_AE, CRF07_BC, CRF08_BC and URFs

HIV-1 genotypes	Counties	Relative risk	Log likelihood ratio	*p*-value	Observed cases			
					Total	Heterosexual contact	Homosexual contact	Intravenous drug injection
CRF01_AE								
Primary Cluster	Dali	17.41	18.1	6.4E-07	10	2 (20.0%)	8 (80.0%)	0
Secondary Cluster	Xishan, Jinning, Anning, Hongta, Jiangchuan, Chengjiang	5.36	8.6	7.0E-03	11	4 (36.4%)	7 (63.6%)	0
CRF07_BC								
Primary Cluster	Wuhua, Panlong, Xishang, Anning	3.68	8.3	1.4E-02	16	7 (43.8%)	7 (43.8%)	2 (12.5%)
CRF08_BC								
Primary Cluster	Gejiu, Kaiyuan, Mengzi, Pingjian, Jianshui, Yuanyang, Jinping, Hekou, Wenshan, Yanshan, Maguan	4.78	41.2	4.4E-16	63	60 (95.2%)	1 (1.6%)	2 (3.2%)
Secondary Cluster	Zhaoyang, Ludian	4.24	13.0	1.5E-04	20	18 (90.0%)	0	2 (10.0%)
URFs								
Primary Cluster	Tenchong, Ruili, Mangshi, Lianghe, Yingjiang, Longchuan	9.62	28.2	4.1E-11	23	14 (60.9%)	0	9 (39.1%)
Secondary Cluster	Kaiyuan, Mengzi, Pingbian, Hekou, Wenshan, Yanshan, Xichou, Maguan, Qiubei	3.51	7.7	2.4E-02	16	16 (100.0%)	0	0

### Genotypic analysis of transmitted drug resistance (TDR)

Among the 411 participants with *pol* sequences, ten (2.4%) were identified as harbouring SDRMs. As shown in Table 3, 0.5% (2/411), 1.2% (5/411), and 0.7% (3/411) of the sequences harboured SDRMs to nucleoside reverse transcriptase inhibitors (NRTIs), non-nucleoside reverse transcriptase inhibitors (NNRTIs), and protease inhibitors (PIs), respectively. As shown in Table 4, the prevalence of SDRMs increased with age (trend χ^2^ = 4.739, *p* = 0.033).

**Table 3:** Demographic characteristics of ten individuals harbouring transmitted drug resistance associated mutations

Sequence ID	Prefecture	Age	Marriage status	Infection route	Genotype	Drug resistance associated mutations		
						NRTI	NNRTI	PI
15R057	Wenshan	47	Unmarried	Heterosexual contact	CRF08_BC	T69D	None	None
15R073	Wenshan	30	Married	Heterosexual contact	BC	None	K103 N, P225H	None
15R089	Wenshan	44	Unmarried	Heterosexual contact	BC	None	K103 N	None
15R091	Kunming	65	Divorced/widowed	Heterosexual contact	CRF07_BC	None	None	F53Y
15R099	Kunming	55	Married	Heterosexual contact	CRF08_BC	K70E	None	None
15R289	Qujing	75	Divorced/widowed	Heterosexual contact	CRF08_BC	None	K103 N	None
15R366	Dali	38	Married	Heterosexual contact	C	None	K101E	None
15R377	Honghe	81	Divorced/widowed	Heterosexual contact	CRF01_AE	None	None	M46I
15R421	Honghe	41	Married	Heterosexual contact	CRF08_BC	None	None	G73S
15DHR38	Dehong	41	Married	Intravenous drug injection	CRF01_AE/BC	None	G190A	None

**Table 4:** The prevalence of SDRMs in different age groups

Age	Total	Subjects with SDRMs	Trend χ2	*p*
< 30	168	1 (0.6%)	4.739	0.033
31–50	165	5 (3.0%)		
≥51	78	4 (5.1%)		

## Discussion

In this study, we performed a cross-sectional HIV-1 molecular epidemiological study to track the characteristics and distribution of HIV-1 genotypes in recent infections in Yunnan. For the first time, the spatial epidemiological characteristics of HIV-1 genotypes among recent infections were analysed.

As described in the previous studies [12], CRF08_BC, CRF07_BC, URFs and CRF01_AE were the main HIV-1 genotypes circulating in Yunnan. However, compared with the surveillance carried out in 2009, the ranks of these four genotypes changed. The proportion of CRF08_BC among recent infections remained the highest and showed no significant difference from 2009 to 2015 (40.8% in 2009, 47.0% in 2014 and 45.3% in 2015; χ^2^ = 1.605, *p* = 0.450) [12, 37]. The proportion of CRF07_BC increased from fourth to second place and showed a statistically increasing trend for 2009–2015 (9.2% in 2009, 14.4% in 2014 and 18.3% in 2015; trend χ^2^ = 8.557, *p* = 0.003). The proportion of URFs declined from second to third place and showed a decreasing trend (27.7% in 2009, 18.2% in 2014 and 18.2% in 2015; χ^2^ = 6.272, *p* = 0.045). The proportion of CRF01_AE declined from third to fourth place and showed a decreasing trend that was not statistically significant (18.5% in 2009, 15.8% in 2014 and 11.4% in 2015; χ^2^ = 5.437, *p* = 0.064). The overall increase in CRF07_BC may be partly due to the increase in CRF07_BC among homosexual contact. Among MSM, the proportion of CRF07_BC increased from 17.2% in 2014 to 42.9% in 2015 (χ^2^ = 5.379, *p* = 0.026).

In addition to the above four predominant genotypes, two subtypes and six CRFs were found in recent infections. The genetic diversity of HIV-1 constitutes a characteristic of the HIV-1 epidemic in Yunnan. HIV-1 recombination was active in the Yunnan-Myanmar border area, where URFs were highly prevalent [9, 38] and more than ten CRFs were identified recently, including CRF57_BC, CRF62_BC, CRF64_BC, CRF65_cpx, CRF78_cpx, CRF82_cpx, CRF83_cpx, CRF86_BC, CRF87_cpx, CRF88_BC and CRF96_cpx [18–25, 39]. In this study, CRF64_BC, CRF83_cpx and CRF87_cpx were found in western Yunnan, which suggested that they were circulating locally. CRF55_01B and CRF59_01B were originally identified in MSM outside Yunnan [15, 16]; CRF85_BC was originally identified in the neighbouring province, Sichuan Province [40]. In this study, these three CRFs were found in eastern Yunnan, where the floating population was relatively larger.

The demographic characteristics associated with HIV-1 genotypes were further analysed using multivariate logistic regression. Because heterosexual contact was the major transmission route (81.1%), the absolute numbers of the main genotypes in this transmission route were the largest. However, the proportions of CRF01_AE and CRF07_BC in homosexual contact were higher than those in heterosexual contact and intravenous drug injection, while the proportion of CRF08_BC in homosexual contact was lower than those in the other two transmission routes. These findings suggested that the homosexually transmitted population remained relatively unique from the other two populations. The proportion of CRF08_BC was higher in females. The reason for this finding could be that the predominant transmission route among the female cases was heterosexual contact (95.8%, 160/167), and in this transmission route, CRF08_BC was the predominant HIV-1 genotype. Furthermore, combined with gender and transmission routes, the subjects were divided into five subgroups. The proportion of CRF08_BC was the highest in the subgroup of female heterosexual contact and the lowest in the subgroup of male homosexual contact (Additional file 6: Table S3). We also found that the proportion of URFs among males was higher than that among females, that the proportion of URFs among non-Chinese was higher than that among Chinese. Usually, URFs were identified in dually or multiply infected individuals. This finding suggested that males and non-Chinese tended to have more complex risk factors. To fully understand HIV-1 genetic characteristics among non-Chinese, the further study should be carried out in the border areas.

For the first time, we described the geographic distribution of HIV-1 genotypes among recent infections at the county level, which was more elaborate than the previous study at the prefecture level [12]. Among the four predominant genotypes, the coverage of CRF08_BC was greater than those of CRF07_BC, URFs and CRF01_AE. However, the other genotypes were only found in one to six counties. In some areas, the four predominant genotypes displayed a clustering tendency. To reveal the spatial clustering characteristics, spatial scanning analysis was performed. There were two statistically significant spatial clusters for CRF08_BC, among which the most likely cluster was located in southeast Yunnan Province and bordered with Vietnam. Strikingly, more than 90% of observed cases in these two spatial clusters were infected through heterosexual contact, which suggested that more efforts for the prevention of heterosexual contact transmission should be taken in these areas.

Among the two statistically significant clusters for CRF01_AE, the most likely cluster in Dali Prefecture and the second secondary cluster in Kunming and Yuxi Prefecture were largely composed of the cases infected through homosexual contact. Among MSM identified as recent HIV infections in this study, the proportions of MSM identified in Kunming and Dali were 46.8% (24/59) and 16.9% (11/59), respectively, and ranked the top two (Additional file 7: Table S4). If the HIV incidence is assumed to be roughly the same among MSM, the population sizes of MSM in Kunming and Dali were the highest in Yunnan Province. Strikingly, the RR of the most likely cluster for CRF01_AE was the highest among all the detected spatial clusters. RR means how many times the estimated risk in cluster is as high as the estimated risk outside the cluster. The higher RR reflects the higher aggregation degree of the specific risk. Thus, this suggested that the risk of CRF01_AE infection displayed a high clustering tendency in this cluster.

As mentioned above, Dehong Prefecture, one of the prefectures bordering Myanmar, was a hot spot of active HIV-1 genetic recombination. In this study, the most likely cluster for URFs was detected in the China-Myanmar border area, including four counties in Dehong Prefecture and one county in Baoshan Prefecture. In this URF cluster, 65.2% (15/23) of the observed cases were Burmese residing in Dehong and Baoshan, and the transmission risks of heterosexual contact (60.9%, 14/23) and intravenous drug use (39.1%, 9/23) coexist. Recent studies also suggested that Burmese living in Yunnan contributed to cross-border transmission of HIV-1 in the China-Myanmar border areas [41, 42]. These results suggested that AIDS prevention and control in border areas should be further considered. The secondary cluster for URFs was located in the nine counties in Honghe Prefecture and Wenshan Prefecture, which almost overlapped with the most likely cluster for CRF08_BC. In this URF cluster, 100% of the observed cases were infected through heterosexual contact, which further suggested the high transmission risk of heterosexual contact in this area. Overall, the spatial clustering analysis for recent infections could provide clues for potential transmission risk, based on which the targeted measures for AIDS control and prevention should be developed.

The prevalence of SDRMs in recent infections was 2.4% in 2015, which was not significantly higher than that in 2014 (1.8%, χ^2^ = 0.263, *p* = 0.791) [37]. However, we found that the prevalence of TDR increased with age. In the group that was above 50 years of age, the prevalence of TDR was over 5% and reached a moderate level, which should be considered. To control HIV-1 TDR, a priority is to strengthen the standard management of patients and regularly perform drug resistance surveillance.

## Conclusions

With the occurrence of HIV-1 epidemics in Yunnan, HIV-1 genotypes have become more diverse. Our study revealed two subtypes, nine CRFs and six discrete URFs circulating in recent infections, among which CRF08_BC, CRF07_BC, URFs, and CRF01_AE were still the predominant strains. The distribution of the four predominant genotypes in the population was associated with demographic characteristics. The geographic distribution of HIV-1 genotypes in recent infections was further analysed at the county level. The four predominant genotypes demonstrated a clustering tendency in some geographic areas, which was confirmed by the spatial scan statistics. Some spatial clusters were associated with a specific transmission route. TDR-associated mutations remained low in recent infections. Our findings provide valuable information to improve strategies to prevent new infections.

## Additional files

Additional file 1: **Table S1.** The comparison of scan statistical results by using different percentages of the population at risk. (PDF 70 kb)

Additional file 2: **Figure S1.** Neighbour-joining phylogenetic tree of the partial *gag* gene. The scale bar indicates 5% nucleotide sequence divergence. Values on the branches represent the percentage of 1000 bootstrap replicates. (PDF 680 kb)

Additional file 3: **Figure S2.** Neighbour-joining phylogenetic tree of the partial *pol* gene. The scale bar indicates 5% nucleotide sequence divergence. Values on the branches represent the percentage of 1000 bootstrap replicates. (PDF 719 kb)

Additional file 4: **Figure S3.** Neighbour-joining phylogenetic tree of the partial *env* gene. The scale bar indicates 10% nucleotide sequence divergence. Values on the branches represent the percentage of 1000 bootstrap replicates. (PDF 591 kb)

Additional file 5: **Table S2.** The constituent of subjects successfully genotyped. (PDF 159 kb)

Additional file 6: **Table S3.** The proportion of CRF08_BC in men and women with different transmission routes. (PDF 68 kb)

Additional file 7: **Table S4.** The distribution of recent HIV infections was attributed to the main transmission routes in each prefecture. (PDF 67 kb)

## Data Availability

The sequences obtained in this study were submitted to NCBI GenBank under accession numbers MH572689-MH573900. The datasets used and/or analysed during the current study available from the corresponding author on reasonable request.

## Abbreviations

BED-CEIA: BED-capture enzyme immunoassay
CRF: Circulating recombinant form
FSW: Female sexual worker
HIV-1: Human immunodeficiency virus-1
IDU: Intravenous drug user
MSM: Men who have sex with men
NNRTIs: Non-nucleoside reverse transcriptase inhibitors
NRTIs: Nucleoside reverse transcriptase inhibitors
PIs: Protease inhibitors
SDRMs: Surveillance drug resistance mutations
TDR: Transmitted drug resistance
URF: Unique recombinant form
