# Supplementary material for: Spatial clusters of HIV-1 genotypes in a recently infected population in Yunnan, China
Source: BMC Infect Dis. 2019 Jul 29;19:669. doi: 10.1186/s12879-019-4276-9 (PMC6664787; doi:10.1186/s12879-019-4276-9)
Supplement: Supplementary file 1 — Table S1. The comparison of scan statistical results by using different percentages of the population at risk. (PDF 70 kb) [file 12879_2019_4276_MOESM1_ESM.pdf]

**Additional file 1: Table S1. The comparison of scan statistical results by using different percentages of the population at risk.**

| Spatial Clusters | | County Code | Number of cases | Relative Risk | Log Likelihood Ratio | *p* -value |
|---|---|---|---|---|---|---|
| **CRF01_AE** | | | | | | |
| 10% of the population at risk | Primary Cluster | 53290100 | 10 | 17.41 | 18.1 | 6.4E-07 |
| | Secondary Cluster | 53012200, 53018100, 53040200, 53011200, 53042100, 53042200 | 11 | 5.36 | 8.6 | 7.0E-03 |
| 30% of the population at risk | Primary Cluster | 53290100 | 10 | 17.41 | 18.1 | 1.3E-06 |
| | Secondary Cluster | 53012200, 53018100, 53040200, 53011200, 53042100, 53042200 | 11 | 5.36 | 8.6 | 1.1E-02 |
| 50% of the population at risk | Primary Cluster | 53290100 | 10 | 17.41 | 18.1 | 2.0E-06 |
| | Secondary Cluster | 53012200, 53018100, 53040200, 53011200, 53042100, 53042200 | 11 | 5.36 | 8.6 | 1.3E-02 |
| **CRF07_BC** | | | | | | |
| 10% of the population at risk | Primary Cluster | 53010200, 53010300, 53011200, 53018100 | 16 | 3.68 | 8.3 | 1.4E-02 |
| 30% of the population at risk | Primary Cluster | 53010200, 53010300, 53011200, 53018100 | 16 | 3.68 | 8.3 | 2.0E-02 |
| 50% of the population at risk | Primary Cluster | 53010200, 53010300, 53011200, 53018100 | 16 | 3.68 | 8.3 | 2.0E-02 |
| **CRF08_BC** | | | | | | |
| 10% of the population at risk | Primary Cluster | 53252200, 53252300, 53250100, 53250200, 53262100, 53253200, 53252800, 53253000, 53262200, 53252400, 53262500 | 63 | 4.78 | 41.2 | 4.4E-16 |
| | Secondary Cluster | 53060200, 53062100 | 20 | 4.24 | 13.0 | 1.5E-04 |
| 30% of the population at risk | Primary Cluster | 53252200, 53252300, 53250100, 53250200, 53262100, 53253200, 53252800, 53253000, 53262200, 53252400, 53262500 | 63 | 4.78 | 41.2 | 3.6E-15 |
| | Secondary Cluster | 53060200, 53062100 | 20 | 4.24 | 13.0 | 2.9E-04 |
| 50% of the population at risk | Primary Cluster | 53252200, 53252300, 53250100, 53250200, 53262100, 53253200, 53252800, 53253000, 53262200, 53252400, 53262500 | 63 | 4.78 | 41.2 | 1.0E-14 |
| | Secondary Cluster | 53060200, 53062100 | 20 | 4.24 | 13.0 | 3.8E-04 |

| | | | | | | |
|---|---|---|---|---|---|---|
| **URFs** | | | | | | |
| 10% of the population at risk | Primary Cluster | 53312300, 53312200, 53312400, 53052200, 53310300, 53310200 | 23 | 9.62 | 28.2 | 4.1E-11 |
| | Secondary Cluster | 53262100, 53262200, 53252300, 53262500, 53252200, 53262300, 53253200, 53262600, 53250200 | 16 | 3.51 | 7.7 | 2.4E-02 |
| 30% of the population at risk | Primary Cluster | 53312300, 53312200, 53312400, 53052200, 53310300, 53310200 | 23 | 9.62 | 28.2 | 1.4E-10 |
| | Secondary Cluster | 53262100, 53262200, 53252300, 53262500, 53252200, 53262300, 53253200, 53262600, 53250200 | 16 | 3.51 | 7.7 | 3.3E-02 |
| 50% of the population at risk | Primary Cluster | 53312300, 53312200, 53312400, 53052200, 53310300, 53310200 | 23 | 9.62 | 28.2 | 2.4E-10 |
| | Secondary Cluster | 53262100, 53262200, 53252300, 53262500, 53252200, 53262300, 53253200, 53262600, 53250200 | 16 | 3.51 | 7.7 | 3.6E-02 |
