# Supplementary material for: Spatial clusters of HIV-1 genotypes in a recently infected population in Yunnan, China
Source: BMC Infect Dis. 2019 Jul 29;19:669. doi: 10.1186/s12879-019-4276-9 (PMC6664787; doi:10.1186/s12879-019-4276-9)
Supplement: Supplementary file 2 — Figure S1. Neighbour-joining phylogenetic tree of the partial gag gene. The scale bar indicates 5% nucleotide sequence divergence. Values on the branches represent the percentage of 1000 bootstrap replicates. (PDF 680 kb) [file 12879_2019_4276_MOESM2_ESM.pdf]

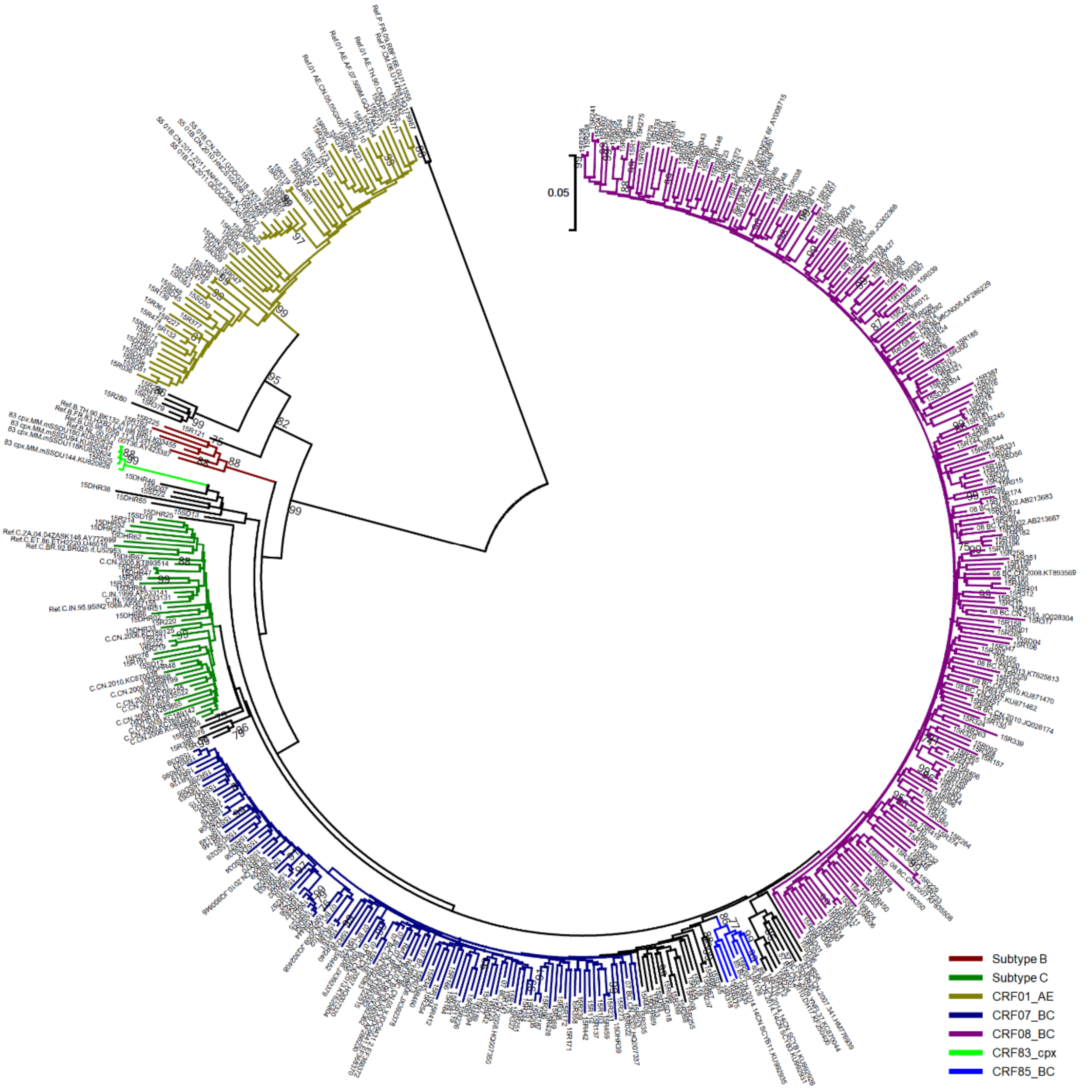

Additional File 2: Figure S1. Neighbour-joining phylogenetic tree of the partial *gag* gene. The scale bar indicates 5% nucleotide sequence divergence. Values on the branches represent the percentage of 1000 bootstrap replicates.
