# Supplementary material for: Spatial clusters of HIV-1 genotypes in a recently infected population in Yunnan, China
Source: BMC Infect Dis. 2019 Jul 29;19:669. doi: 10.1186/s12879-019-4276-9 (PMC6664787; doi:10.1186/s12879-019-4276-9)
Supplement: Supplementary file 5 — Table S2. The constituent of subjects successfully genotyped. (PDF 159 kb) [file 12879_2019_4276_MOESM5_ESM.pdf]

Additional file 5: Table S2. The constituent of subjects successfully genotyped.

Characteristics	BED positive subjects	Subjects obtaining suptypes	$\chi 2$	*P*
**Total**	586	439		
**Prefectrue**			17.889	0.269
Baoshan	27	23		
Chuxiong	9	7		
Dali	37	31		
Dehong	71	30		
Diqing	3	0		
Honghe	83	63		
Kunming	79	60		
Lijiang	17	15		
lincang	28	19		
Nujiang	3	3		
Puer	32	27		
Qujing	39	30		
Wenshan	64	58		
Xishuangbanna	13	3		
Yuxi	29	25		
Zhaotong	52	45		
**Gender**			0.114	0.746
Male	357	272		
Female	229	167		
**Nationality**			2.287	0.154
Chinese	543	417		
Non-Chinese	43	22		
**Age**			1.387	0.926
≤20	55	38		
21-30	191	136		
31-40	132	96		
41-50	109	86		
51-60	42	32		
⩾61	57	51		
**Race/ethnicity**			6.053	0.419
Han	345	272		
Yi	48	34		
Dai	41	18		
Hani	30	23		
Jingpo	23	12		
Zhuang	21	21		
Others	78	59		
**Marital Status**			0.321	0.851
Unmarried	198	148		
Married	264	192		
Divoiced/Widowed	124	99		
**Infection Routes**			2.517	0.286
Heterosexual contact	462	354		
Homosexual contact	59	49		
Intravenous drug injection	65	36		
