# Supplementary material for: Spatial clusters of HIV-1 genotypes in a recently infected population in Yunnan, China
Source: BMC Infect Dis. 2019 Jul 29;19:669. doi: 10.1186/s12879-019-4276-9 (PMC6664787; doi:10.1186/s12879-019-4276-9)
Supplement: Supplementary file 6 — Table S3. The proportion of CRF08_BC in men and women with different transmission routes. (PDF 68 kb) [file 12879_2019_4276_MOESM6_ESM.pdf]

Additional file 6: Table S3. The proportion of CRF08_BC in men and women with different transmission routes.

	Contact history	Total	CRF08_BC	The proportion of CRF08_BC	*p*	OR (95% CI)
Men	Heterosexual contact	194	89	45.9%	-	1.000
	Homosexual contact	49	3	6.1%	<0.001	0.077 (0.023~0.256)
	Intravenous drug injection	29	11	37.9%	0.424	0.721 (0.323~1.607)
Women	Heterosexual contact	160	93	58.1%	0.022	1.638 (1.073~2.498)
	Intravenous drug injection	7	3	42.9%	0.875	0.885 (0.193~4.059)
