# Supplementary material for: Spatial clusters of HIV-1 genotypes in a recently infected population in Yunnan, China
Source: BMC Infect Dis. 2019 Jul 29;19:669. doi: 10.1186/s12879-019-4276-9 (PMC6664787; doi:10.1186/s12879-019-4276-9)
Supplement: Supplementary file 7 — Table S4. The distribution of recent HIV infections was attributed to the main transmission routes in each prefecture. (PDF 67 kb) [file 12879_2019_4276_MOESM7_ESM.pdf]

Additional file 7: Table S4. The distribution of recent HIV infections was attributed to the main transmission routes in each prefectures.

Prefectrue	Heterosexual contact	Homosexual contact	Intravenous drug injection
Baoshan	20	3	4
Chuxiong	9	0	0
Dali	24	11	2
Dehong	39	2	30
Diqing	3	0	0
Honghe	78	3	2
Kunming	44	24	11
Lijiang	14	1	2
lincang	24	1	3
Nujiang	3	0	0
Puer	30	1	1
Qujing	36	2	1
Wenshan	60	4	0
Xishuangbanna	10	2	1
Yuxi	20	4	5
Zhaotong	48	1	3
**Total**	462	59	65
